# The Role of Sperm Centrioles in Human Reproduction – The Known and the Unknown

**DOI:** 10.3389/fcell.2019.00188

**Published:** 2019-10-01

**Authors:** Tomer Avidor-Reiss, Matthew Mazur, Emily L. Fishman, Puneet Sindhwani

**Affiliations:** ^1^Department of Biological Sciences, College of Natural Sciences and Mathematics, The University of Toledo, Toledo, OH, United States; ^2^Department of Urology, College of Medicine and Life Sciences, The University of Toledo, Toledo, OH, United States

**Keywords:** centriole, sperm, embrio, cilium, centrosome, infertility, male factor, reproduction

## Abstract

Each human spermatozoon contains two remodeled centrioles that it contributes to the zygote. There, the centrioles reconstitute a centrosome that assembles the sperm aster and participate in pronuclei migration and cleavage. Thus, centriole abnormalities may be a cause of male factor infertility and failure to carry pregnancy to term. However, the precise mechanisms by which sperm centrioles contribute to embryonic development in humans are still unclear, making the search for a link between centriole abnormalities and impaired male fecundity particularly difficult. Most previous investigations into the role of mammalian centrioles during fertilization have been completed in murine models; however, because mouse sperm and zygotes appear to lack centrioles, these studies provide information that is limited in its applicability to humans. Here, we review studies that examine the role of the sperm centrioles in the early embryo, with particular emphasis on humans. Available literature includes case studies and case-control studies, with a few retrospective studies and no prospective studies reported. This literature has provided some insight into the morphological characteristics of sperm centrioles in the zygote and has allowed identification of some centriole abnormalities in rare cases. Many of these studies suggest centriole involvement in early embryogenesis based on phenotypes of the embryo with only indirect evidence for centriole abnormality. Overall, these studies suggest that centriole abnormalities are present in some cases of sperm with asthenoteratozoospermia and unexplained infertility. Yet, most previously published studies have been restricted by the laborious techniques (like electron microscopy) and the limited availability of centriolar markers, resulting in small-scale studies and the lack of solid causational evidence. With recent progress in sperm centriole biology, such as the identification of the unique composition of sperm centrioles and the discovery of the atypical centriole, it is now possible to begin to fill the gaps in sperm centriole epidemiology and to identify the etiology of sperm centriole dysfunction in humans.

## Introduction

**FIGURE 1:**
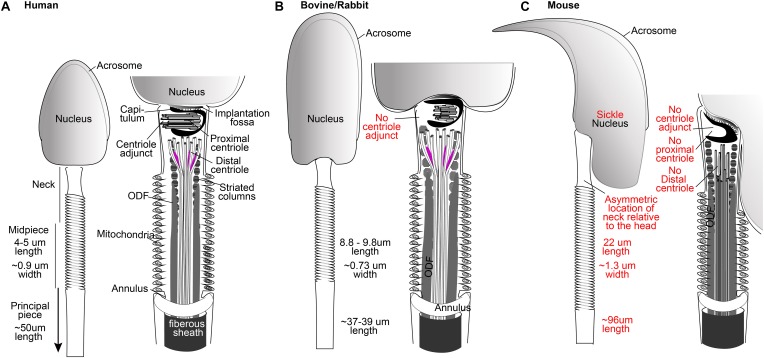
The spermatozoa of human **(A)**, bovine, and rabbit **(B)** share many properties not found in mice **(C)**, including the presence of one typical and one atypical centriole. Drawings are based on electron microscopy of Zamboni and Stefanini (1971), Fawcett (1975), and Ounjai et al. (2012). Mammalian sperm dimensions are from Cummins and Woodall (1985).

Centrioles are essential for animal development and physiology, as demonstrated by a variety of experiments that have tested the centriole’s role directly (Bettencourt-Dias et al., 2011). Most of these experiments have been performed *in vitro*, in human immortalized cells, or in animal models, limiting our knowledge of the centriole’s role in human reproduction. In general, the centriole’s role is expected to be similarly essential in humans because of its conservation throughout animal evolution (Carvalho-Santos et al., 2011). However, the centrioles of sperm and the early embryo of murine animals are exceptions to this evolutionary conservation (Figure 1). While humans and many other mammals have centrioles in their spermatozoa and early embryos, mice, rats, and hamsters (the most common experimental mammals) do not have recognizable centrioles in their spermatozoa and early embryos (Schatten et al., 1986; Sathananthan et al., 1996; Phillips et al., 2014). These major differences in centriole appearance raise the question: *What exactly is the role of the centriole in human fertilization and early embryonic development?*

One extreme and unlikely idea is that sperm centrioles are not needed in humans because they are undetectable in mice sperm and appear to be expendable for murine live birth. However, an alternative hypothesis is that mice have evolved a novel biology of sperm and early embryonic centrioles, and, therefore, studying their role in reproduction is not applicable to their role in human reproduction and their clinical implication. Indeed, all studied non-murine mammals *do* have centrioles in their sperm and early embryo, and, in certain animals, some evidence indicates that these centrioles are necessary. Here, we will explore this evidence and the fact that the sperm of humans and non-murine mammals have two centrioles (one with typical structure and one atypical), not one, as was concluded in the past (Avidor-Reiss and Fishman, 2018). We start with a brief general introduction to centrioles, then progress to discussion of sperm and early embryonic centrioles. Then, we describe clinical studies that implicate the centriole in human reproduction. Finally, we propose future directions to resolve the question of the role of the sperm centriole role in the embryo. This review focuses on the role of the mature sperm (spermatozoon) centrioles in the zygote, and it does not address the role of centrioles in germline (spermatogonia, spermatocyte, and meiosis) development [reviewed in Riparbelli and Callaini (2011)].

## Most Dividing Animal Cells Have Precisely Two Centrioles with a Conserved Barrel Shape

Centrioles are evolutionarily conserved cellular components essential for fertilization, cell development, and animal physiology through their function in the cell [reviewed in Nigg and Raff (2009)]. The centriole is a cylinder built from nine triplet microtubules arranged in a ring-like formation to form a barrel structure. In the centrosome, the centrioles are surrounded by pericentriolar material (PCM) (Vorobjev and Chentsov Yu, 1982) [reviewed in Lange and Gull (1996), Mennella et al. (2012)]. In cilia and flagella, the centriole triplets are continuous with the doublet microtubules of the axoneme. Centrioles are composed of more than one hundred proteins (Preble et al., 2000) [reviewed in Winey and O’Toole (2014), Nigg and Holland (2018)], and, while new components of centriolar protein structure continue to be discovered, determining their functionality can be a challenge, both because they often have essential roles in early development [reviewed in Schatten and Sun (2010)], and because their function may vary from one cell type to another [previously reviewed in Loncarek and Bettencourt-Dias (2018)].

Most cells have two centrioles during early interphase. Most centrioles form by “duplication,” where each of the two-preexisting centrioles direct the formation of one new procentriole, providing a mechanism to control the number of centrioles formed. Centrioles may infrequently form *de novo*, in the absence of preexisting centrioles or in specialized cell types, but often times, this mechanism results in too many centrioles being formed (Loncarek and Khodjakov, 2009; Shahid and Singh, 2018). Progression from a procentriole to a mature centriole correlates with cell-cycle progression (Lange and Gull, 1996). A pair of centrioles is made of the cell’s older mother centriole and a younger daughter centriole. As a result, the number of centrioles in the cell oscillates between two centrioles during early interphase and four centrioles during late interphase and mitosis (Fukasawa, 2007).

Centrioles are integral to the cilia, which confer motility and extra-cellular communication potential to the cell. In most cell types, the cilium is formed by the mature centriole within the cell, which is known as the basal body [reviewed in Avidor-Reiss and Gopalakrishnan (2013)]. However, it was recently proposed that in sperm, the flagellum is formed by the younger daughter centriole, but this proposal requires further support (Garanina et al., 2019). Centrioles are also involved in the cell division process, but their contributions vary in distinct cell types. In general, centrioles recruit PCM to form a centrosome. This centrosome associates with microtubules to assemble an aster that localizes to the spindle poles during mitosis. The asters’ main function is to determine the axis of cell division and the number of spindle poles (Bobinnec et al., 1998). Interestingly, some animal cells [oocytes, importantly (Calarco et al., 1972)] and many plant cells (Joshi and Palevitz, 1996) lack centrioles. This implies that centrioles are not necessary in all cell types and that some cells can successfully complete mitosis and cellular organization without centriolar influence. Therefore, the significance of centrioles may vary in the several cell types that normally contain them.

## Human Mature Oocytes Have No Centrioles

Oocytes contain most of the elements necessary for zygotic development (i.e., Golgi, ER, and proteasomes), including centriolar and PCM proteins, but they lack assembled centrioles. Oocyte centrioles are eliminated, either by extrusion or inactivation, during oogenesis in humans and most studied animals (Hertig and Adams, 1967; Sathananthan et al., 1985; Schatten et al., 1986; Pickering et al., 1988; Crozet, 1990; Navara et al., 1994; Crozet et al., 2000) [reviewed in Sathananthan et al. (2006)]. Limited studies have explored the mechanism of oocyte centriole elimination, but they propose a mechanism reliant on some of the same proteins that are involved in centrosome biology, such as PLK1 (Pimenta-Marques et al., 2016). Centriole elimination by the oocyte necessitates contribution of both spermatozoan centrioles to the zygote to ensure an appropriate number of centrioles in the embryo cell (Connolly et al., 1986; Schatten, 1994; Manandhar et al., 2005; Pimenta-Marques et al., 2016). More information can be found in a recent review on how animal oocytes assemble spindles in the absence of the centriole at Severson et al. (2016).

## Mouse Spermatozoon and Zygote Have No Recognizable Centrioles

In mice, centriole inheritance to the embryo diverges from other mammalian models. In mice, centrioles appear to completely degenerate during spermiogenesis (Manandhar et al., 1998), and, as a result, sperm centrioles may not be present within the zygote (Schatten, 1994; Hewitson et al., 1997) (Figure 1C). It has been proposed that zygotic centrioles are inherited maternally or form *de novo* (Schatten et al., 1985, 1986, 1991; Gueth-Hallonet et al., 1993; Hewitson et al., 1997). The support for non-paternal inheritance is based on several observations, including: (1) zygotes do not appear to have a dominant sperm aster and, instead, have maternal mini-asters, (2) the sperm axoneme is present in the oocyte but lacks microtubules and shows no association with mitotic poles, (3) centrioles are observed only starting in the 32/64 cell stage of early embryos (Gueth-Hallonet et al., 1993), and (4) intracytoplasmic sperm injection (ICSI) with disassociated sperm nuclei is sufficient for embryo development (Kuretake et al., 1996; Yan et al., 2008). The question of *why* the centrioles of the murine zygote are not inherited from the sperm, as is seen with other mammals, remains unanswered.

## Parthenogenic Cells Have an Unregulated Number of Centrioles

The ability to form a parthenogen (an embryo that is developed from an unfertilized egg that lacks centrioles) and parthenogenic cell lines is often referenced to suggest that sperm centrioles are not essential in mammals. However, it is important to note that mammalian parthenogenesis does not lead to viable offspring (Wininger, 2004). It has been proposed that imprinting defects are the main barrier to viability in mammalian parthenogenesis (Kono, 2006; Miller et al., 2019), but concomitant centriole abnormalities present during mammalian parthenogenesis may also contribute to this barrier.

The importance of sperm centrioles in mammalian parthenogenic embryos is apparent, in that parthenogenic cells have an abnormal number of centrioles, as expected from a *de novo* mechanism of centriole formation (Brevini et al., 2012). In the absence of preexisting centrioles (as would be expected in parthenogenesis), an unregulated number of centrioles is expected to form *de novo* in the activated oocytes or subsequent cell types of the embryo (Hinchcliffe, 2011). The presence of too many or too few centrioles, as is observed in mammalian parthenogenic cell lines, can lead to chromosomal instability and, ultimately, cell death (Sir et al., 2013; Godinho and Pellman, 2014). These centriole abnormalities can partially explain the presence of high levels of chromosomal abnormalities in parthenogenic embryos and their ultimate inability to develop viable offspring (Bhak et al., 2006). Therefore, it is possible that non-murine zygotes need two centrioles to produce a viable embryo, and further study of parthenogenic centrioles is necessary, particularly to determine at what stage they form, how many are formed, and how they function.

## Most Mammalian Spermatozoa and Zygotes Have Centrioles

### The Spermatozoon Neck Has One Typical and One Atypical Centriole

The spermatozoon is a streamlined, motile cell that is made of a head and tail and a neck that contains the two centrioles (Figure 1). The head carries half of the genetic material of the embryo, along with proteolytic proteins within the acrosome that help in reaching the oocyte (Cox et al., 2002; Yoon et al., 2008). The tail is a specialized cilium that propels the sperm cell to meet the egg (Basiri et al., 2014; Avidor-Reiss and Leroux, 2015). Spermatozoon morphology is variable across mammals due to evolutionary pressure, making it one of the most diverse cells when compared with the corresponding cell type in other species (Gomendio and Roldan, 2008; Breed et al., 2014). Importantly, a major difference is exhibited between human and mouse spermatozoa. While human sperm, like that of most other mammals, is morphologically broad and flat (spatula-shaped), mouse sperm is curved, long, and narrow (sickle-shaped) (Figure 1A). In humans and most other mammals, the tail is attached to the head near its center, but in mice, the attachment is to the side of the head (Figure 1C). Also, the tail dimensions are very similar between humans and other mammals, but the mouse tail is about twice the length of that of humans. Mice exhibit substantial evolutionary conservation of many of the critical developmental processes in other mammals and, in general, are a beneficial model. However, the differences described above suggest that some aspects of murine sperm have evolved differently than and away from other mammals, including humans. Therefore, translating information on this topic from mice to humans requires caution.

The human sperm neck contains two centrioles as well as a specialized PCM. The proximal centriole (PC) is found just near the head base, and the distal centriole (DC) is located further from the head, attached to the base of the axoneme (Figure 1A). During sperm formation, the PC forms a centriolar adjunct that is thought to organize the development of the neck region and to guide the manchette to form the axis of nuclear flattening (Fawcett and Phillips, 1969; Lehti and Sironen, 2016). At the same time, the DC microtubules extend to form the axonemal microtubules of the tail (Fawcett and Phillips, 1969). The centrioles become embedded in structural material, including the capitulum around the PC, the striated columns filling most of the neck, and the outer dense fibers (ODFs) (Figure 1A). The capitulum and the striated columns form a specialized PCM that also contains centrosomal proteins (Fawcett and Phillips, 1969; Fishman et al., 2018).

The mature spermatozoon obtains its unique streamlined morphology and composition during spermiogenesis [reviewed in Avidor-Reiss and Fishman (2018)]. Early spermatids contain both a PC and a DC with typical centriole structure and composition. The fate of the DC and PC during spermatid differentiation into spermatozoa varies according to animal species, but both centrioles are present in mature spermatozoa of humans and most other mammals. The “centriole remodeling” that occurs during differentiation leaves one centriole that maintains a typical structure (the PC) and one that obtains an atypical structure (the DC). This structure consists of splayed microtubules doublets and centrosomal proteins but maintains the ability to function in the zygote (Fishman et al., 2018). What remains unknown is the usefulness, unique function, and fate in the embryo of these remodeled centrioles and specialized PCM.

### The Two Sperm Centrioles Function in Most Mammalian Early Embryos

Most dividing cells require two centrioles, each of which localizes to one of the spindle poles that mediate chromatid separation between daughter cells when the cell divides. The same is expected for the first cell of the embryo (the zygote). Also, immediately after sperm-egg fusion, the sperm centrioles form an aster, and, shortly thereafter, the two sperm centrioles undergo “duplication,” forming two new daughter centrioles [reviewed in Avidor-Reiss and Fishman (2018)]. Finally, at some point in embryonic development, centrioles are expected to template a primary cilium. While the timing for this is unknown in humans and non-murine animals, in mice, the first primary cilia have been observed in blastocysts with 64–100 cells, only after implantation on epiblast cells (Bangs et al., 2015). Based on these functions, sperm centrioles are widely expected to be essential in the embryo for development.

In many animals, such as worms and fish, centrosome reduction results in the elimination of PCM proteins, with little or no apparent change to centriole structure. In insects, the two centrioles are modified, one slightly and one so dramatically that it was only recently discovered (Gottardo et al., 2015; Khire et al., 2016; Dallai et al., 2017; Fishman et al., 2017). The oocyte contains appropriate PCM material that joins with the spermatozoan centrioles after they are introduced. The necessity of both sperm centrioles and maternal PCM for embryo development has been demonstrated in flies (Blachon et al., 2014), nematodes [reviewed in Leidel and Gonczy (2005)], and fish (Yabe et al., 2007). These publications suggest that in many animals, remodeled sperm centrioles are essential in the embryo, and, therefore, the lack of an integral role for sperm centrioles in mice is an exception.

The paternal inheritance of the PC in the zygote has been well established in several mammalian models. The presence of sperm centrioles in the zygote has been demonstrated in cows (Navara et al., 1994), sheep (Le Guen and Crozet, 1989), primates (Simerly et al., 1995; Wu et al., 1996), and pigs (Kim et al., 1996). However, the essential functions of zygotic centrioles in these models have not been directly demonstrated. Remodeling of zygotic centrioles has been established through ultrastructural and some immunological studies in cows (Long et al., 1993; Navara et al., 1994; Sutovsky et al., 1996; Sutovsky and Schatten, 1997), sheep (Le Guen and Crozet, 1989; Crozet, 1990; Crozet et al., 2000), rabbits (Longo, 1976; Szollosi and Ozil, 1991; Yllera-Fernandez et al., 1992; Pinto-Correia et al., 1994; Terada et al., 2000), and cats (Comizzoli et al., 2006). More recently, it was shown that cow zygotes also inherit a second atypical centriole (the DC) (Fishman et al., 2018). This inheritance pattern suggests that centrioles *may* play an important role in the developing zygote; however, it is unclear exactly *what* and *how* these centrioles may be contributing.

## The Zygote of Humans Inherits Two Functional Sperm Centrioles

Though studies in humans are lacking, two sperm centrioles are likely present in the early zygotes and four during mitosis. The human zygote inherits the PC from sperm, which is well established (Sathananthan et al., 1991). Also, the human zygote is likely to inherit the DC because (1) the spermatozoa have a remodeled DC that is attached to the axoneme (Fishman et al., 2018), and (2) the base of the axoneme is located at one of the spindle poles (Asch et al., 1995; Van Blerkom, 1996; Simerly et al., 1999; Kovacic and Vlaisavljevic, 2000).

Kai et al. (2015) showed the presence of two centrosomes in zygotes with two pronuclei (presumably fertilized by a single sperm) by immunofluorescence staining for centrosomes. Sathananthan et al. (1991) identified centrioles within the centrosomes of zygotes with two pronuclei by electron microscopy following *in vitro* fertilization; two centrosomes with one or two centrioles were observed. Both Kai et al. (2015) and Sathananthan et al. (1991) have observed four centrosomes in early zygotes with three pronuclei, suggesting that dispermic embryos provide extra centrioles that form extra centrosomes. Interestingly, only three and not four spindle poles were observed in these zygotes. The presence of only three poles in dispermic embryos, despite the presence of four centrosomes, raises the question of whether the number of poles is determined by the sperm centrioles or pronuclei. However, these studies are consistent with the idea that human sperm normally contribute two centrioles to the zygote.

After membrane fusion, the centrosome forms one sperm aster near the head, which then enlarges throughout most of the zygote cytoplasm (Van Blerkom, 1996; Simerly et al., 1999). During pronuclear migration, this aster splits into two. Later, each aster localizes to one pole of the forming bipolar mitotic spindle. Interestingly, after initial formation, the asters collapse and are either small or unrecognizable during metaphase. This collapse is transient, as the asters reappear in anaphase. Why asters collapse during metaphase is unknown and needs further investigation.

It is commonly thought that human sperm centrioles are essential for pronuclear migration, based on studies on abnormal sperm asters in the zygote. Immunofluorescence analysis of fertilized zygotes with arrested development has shown disorganized and diminished sperm aster microtubule arrays, along with the lack of pronuclear formation and/or migration (Asch et al., 1995; Van Blerkom, 1996). These concurrent findings suggest that the sperm aster may be responsible for normal pronuclear development. These studies are limited by the fact that microtubules are also nucleated by non-centriolar microtubule-organizing centers, and that, in many of these studies, a defect in the centriole was not found or studied, making the specific role of the centriole and sperm aster in the zygote unclear.

After entering the egg, the PC is thought to be released from the sperm neck structures before recruiting PCM and forming astral microtubules, a process proposed to be mediated by proteasomes (Wojcik et al., 2000; Rawe et al., 2008; Sutovsky, 2018), but it is unknown if the DC is detached from the neck structures. Spermatozoan proteasomes have been localized to the neck region/midpiece in close association with the centrioles; however, relatively little is known about their function. Spermatozoa from humans and bovine, preloaded with function-blocking anti-proteasome antibody, resulted in disrupted sperm aster formation and pronuclear development/apposition of human oocytes, despite the lack of observable centriole structural deficits (Rawe et al., 2008). This suggests that spermatozoan proteasomes may play an important role in centriole contribution to zygote development. More pharmacological and genetic studies should be done to investigate the mechanism and the precise role of the proteasome in sperm and zygote centrioles.

## Epidemiological Evidence for Centriole Contribution to Human Fertility

Sperm with impaired centrioles were long thought to result in embryos with abnormal cleavage and infertility (Sathananthan, 1994; Schatten, 1994). This idea is now widely accepted and is supported by some epidemiological studies using artificial insemination to overcome barriers to fertilization in defective sperm samples. However, this idea is based on few studies, which analyzed 1–10 patients and few fertile donors as a control (Table 1). The phenotypes observed in these studies are diverse, providing opportunity for many inferences but making direct correlation with centriole phenotype difficult. Also, many studies are complicated by the presence of other defects in the sperm (in addition to the centriole), which may have a significant impact on the embryo phenotype. In particular, one can expect that some forms of centriolar defect may originate early in germline development and will result in abnormal DNA content due to abnormities in mitosis or meiosis in addition to the centriole defect, as was found for the mutation in the centriolar protein Centrobin (Liška et al., 2009). Unfortunately, these drawbacks negatively impact the confidence that we have in inferring the role of the sperm centriole in embryo development. However, the totality of these studies suggests that sperm centrioles are likely essential for normal embryo development.

**TABLE 1:** Parameters of various studies of direct sperm phenotype dysfunctions.

**Study**	**Male patient (n)**	**Female partner**	**Control men (n)**	**Sperm morphology and method**	**Total sperm count (million)**	**Sperm volume (mL)**	**Sperm concentration (million/mL)**	**Sperm motility (%)**	**Progressive motility (%)**	**Sperm viability (%)**
Normal sperm reference values World Health Organization [WHO], 2010				>4	>39	>1.5	>15	>40	>32	>58
Nakamura et al., 2002	1 man 38-year-old		2	90% **Globozoospermic:** IF		2.5	12	41.7		
Chemes et al., 1999	10 sterile men			**Head-neck defect**: TEM; nine patients had 75–100% spermatozoa with minute cephalic ends and 0–25% abnormal head-middle piece attachments.			Oligozoospermia	Ranged from normal to severe asthenozoospermia (3–60% total motility		
Rawe et al., 2002	1 man 36-year-old	31-year-old, normal ovarian function	1	**Head-neck defect**: TEM				Total asthenozoospermia		
Porcu et al., 2003	2 brothers #1: 25 and #2: 36-year-old	#1: 24-year-old, irregular menses, normal hysterosalpingography and hormonal assessment #2: 31-year-old, regular menses, normal hysterosalpingography and hormonal assessment	2	#1: 100% **teratozoospermia**; 71% tail-less heads #2: 98–100% **teratozoospermia:** TEM + IF; 45–83% of isolated heads; 6–29% with head-tail misalignment;	#1:		#1: 1–1.7 normal sperm + 7.9–16.8 acephalic sperm #2: 4.5 normal sperm); + 0.5 tail-less heads) + 4.5–34 isolated motile tails	#1: 15–30 #2: 10–20	#1: no progressive motility #2: no progressive motility	
Emery et al., 2004	1 man 50-year-old	45-year-old, possible decreased ovarian reserve	1	**Easily decapitated sperm:** TEM; 23% normal head forms and 2% normal tail forms		2.5	33		13	
Sha et al., 2017	1 man 30-year-old		few	**MMAF:** Teratozoospermia		2.9	20	1.8 Asthenozoospermia		
Fonttis et al., 2002	1 man 39-year-old	31-year-old, healthy with no prior pregnancy	0	13% normal; 93% axonemal alterations (TEM); 90% abnormal mitochondrial sheaths (IF)		3	64^6^	15 **Asthenozoospermia;**	5	
Terada et al., 2009	1 man 35-year-old	31-year-old, normal fertility	3	normal	12	2	6	27 **Oligoasthenozoospermia**		
Moretti et al., 2019	1 man-year-old		2	**Globozoospermia**						
Garanina et al., 2019	2 men #1 41-year-old #2 34-year-old	Normal	5	**Unexplained**; normal	Normal	Normal	Normal	Normal	Normal	Normal
Moretti et al., 2017	1 man 37-year-old	35-year-old Normal	1	55% **head-tail defect**: TEM + IF; 26% completely detached heads; 19% isolated tails		2.5–3.5	12–16	0		

*IF, immunofluorescence; TEM, transmission electron microscopy.*

Intracytoplasmic sperm injection is a useful treatment for male infertility due to interference with sperm translocation to the egg and fusion with it, but not for infertility due to dysfunction of sperm components that are needed after fusion (Palermo et al., 1992). Indeed, ICSI allows successful fertilization for many couples, but this may require multiple attempts, and, in many other couples, this fails [reviewed in Neri et al. (2014)]. This suggests that in these failed cases, infertility may be caused by processes downstream of gamete fusion that depend on the male contribution. However, the multifactorial nature of reduced semen quality has prevented many of the underlying contributory molecular mechanisms from being fully elucidated.

The only routine clinical assay to assess sperm phenotype is semen analysis, and this assay by itself cannot implicate the sperm centriole in infertility. Semen analysis compares the quality of the patient’s semen and sperm against several standardized parameters and helps identify specific defects in the male which are seen in up to one-third of infertile couples (Isidori et al., 2006) (Table 1). One group of sperm defects with several phenotypes that could originate from centriolar defect is idiopathic male factors (male infertility due to a sperm defect that has no explanation, i.e., when the male is infertile despite having normal semen analysis, history, and physical examination, and when female factor infertility has been ruled out) (Chemes and Rawe, 2010). For example, it was suggested that abnormal centrioles will form an abnormal sperm tail (which is one phenotype of teratozoospermia, defined as presence of sperm with abnormal morphology), a defective sperm axoneme resulting in poor motility (asthenozoospermia), or a combination of the two (asthenoteratozoospermia). Some of these cases of male factor infertility may be due to a subtle sperm centriolar defect–a hypothesis that will be examined in the following sections.

### Asthenoteratozoospermia

Extremely abnormal sperm morphology can inhibit spermatozoan movement through the female reproductive tract and prevent fusion with the oocyte. These morphological abnormalities have many causes and take many forms, and their definition is controversial, but they can be present in ∼10% of infertile men (Khan et al., 2011). As described below, some of these cases are suggested to be caused by or accompanied by a centriolar defect, but these defects have not been fully elucidated.

#### Head-Neck Defect

Head-neck defects characterized by separated heads and tails provide the strongest evidence that human centrioles are essential for fertility; however, this evidence does have some limitations. In mice, injection of the sperm nucleus without the sperm tail results in a fertilized egg that produces offspring, suggesting that mouse sperm centrioles are dispensable (Kuretake et al., 1996). In contrast, in humans, injection of the sperm nucleus without the sperm tail is insufficient to produce offspring (Palermo et al., 1997; Emery et al., 2004). This may suggest that centriolar absence is the reason for infertility, but this interpretation is limited by the fact that other components in the tail may be essential for embryo development in humans. For example, it was recently shown that MicroRNA-34c localizes to the sperm neck and is required for the first cleavage division (Liu W.-M. et al., 2012; Fereshteh et al., 2018). Therefore, it is not possible in these types of experiments to disentangle the phenotypical consequences of not providing paternal centrioles from that of not delivering other factors essential in the zygote.

**FIGURE 2:**
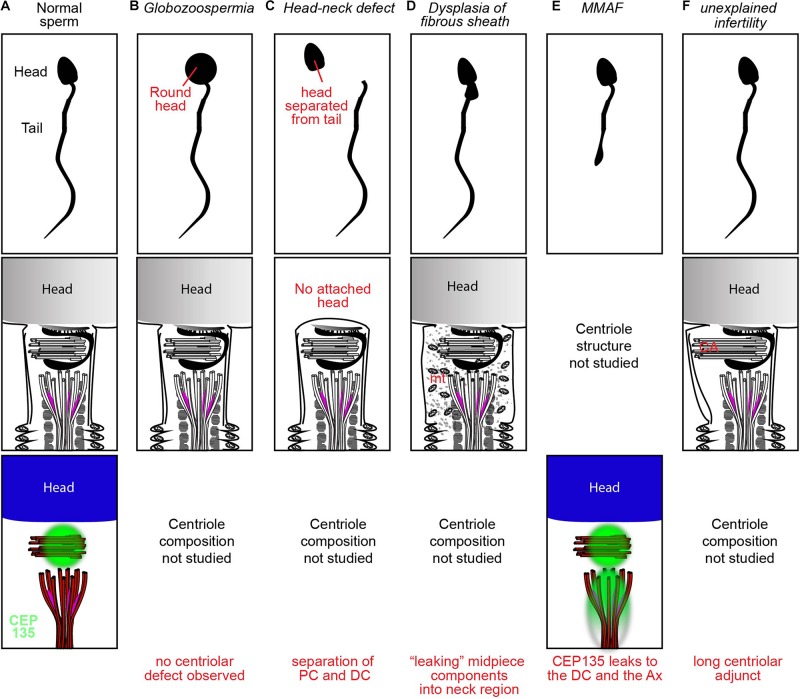
Examples of spermatozoan morphology, structure, and protein distributions in a fertile donor **(A)**, oligospermia **(B)**, head-neck defect **(C)**, DFS **(D)**, MMAF **(E)**, and unexplained infertility **(F)**.

The centriole-containing sperm neck is the mechanically weakest part of the sperm. Externally applied pressure may cause the sperm neck to break, forming two fragments: a head fragment and a neck-tail fragment (Firat-Karalar et al., 2014). Indeed, decapitated sperm often breaks between the head and tail and only more rarely at the midpiece (Baccetti et al., 1989; Rawe et al., 2002; Porcu et al., 2003; Gambera et al., 2010). Another rare form of break is caused by disassociation between the PC and DC (Holstein et al., 1986). A variety of structural abnormalities have been observed in sperm with head-neck defect, but their causes are still not well understood. Easily decapitated sperm syndrome is one of the mildest subtypes of head-neck defect teratozoospermia, as some heads and tails in the ejaculate remain connected (Perotti et al., 1981; Toyama et al., 2000). Electron microscopy of decapitated sperm often reveals an absent basal plate and/or implantation fossa with observable breaks between the head and tail (Baccetti et al., 1989) (Figure 2). Injection of individual or separated sperm parts allows oocyte activation and pronucleus formation but does not facilitate pronuclear migration and fusion, leading to abnormal embryos (Colombero et al., 1996). Embryos injected with dissected isolated sperm tails or separated heads and tails show chromosome mosaicism, suggesting centrosome and centriole dysfunction (Palermo et al., 1997). However, overall, ICSI has mixed outcomes with easily decapitated sperm, sometimes overcoming infertility but many other times failing despite good embryo morphology (Kamal et al., 1999; Saias-Magnan et al., 1999).

Chemes et al. (1999; Table 1) used electron microscopy of testicular biopsies from patients with this defect to show that the forming sperm tail developed uncharacteristically independent of the nucleus, a phenomenon which can be caused by a centriolar defect. Rawe et al. (2002; Table 1) used immunofluorescence of human spermatozoa with this defect injected into bovine oocytes to show scarce centrosome-associated microtubules and arrested sperm asters, as compared to fertile donor controls that formed sperm asters in a majority of samples. Ejaculated spermatozoa from patients were also injected into human metaphase II oocytes and analyzed by immunofluorescence. No pronuclei fusion or zygote cleavage occurred with the first attempt. The following three attempts selectively injected sperm with near-normal head-neck alignment; they resulted in fusion and cleavage, but the embryos failed to develop into pregnancy. Emery et al. (2004; Table 1) performed ICSI with easily decapitated sperm and reported a successful birth when the separated tails were placed immediately next to the sperm head within the oocyte. The authors surmised that careful integration of all parts of the sperm (head and tail) during ICSI ensured that centrioles, which are likely lost in other less delicate techniques, are included and ultimately allow normal development of the oocyte. Indeed (Table 1), successful pregnancies can be achieved by ICSI using intact sperm from men with abnormal head-tail junction (Porcu et al., 2003; Gambera et al., 2010).

#### ICSI With Sperm Tail Abnormality (MMAF)

Multiple morphological abnormalities of the flagella (MMAF) is a rare form of asthenoteratozoospermia known to cause male infertility (Wang et al., 2019). MMAF is caused by mutations in proteins of the axoneme or transport the mechanism that forms it [e.g., inner-arm heavy chain DNAH1 (Ben Khelifa et al., 2014), or the intraflagellar transport (IFT)-associated protein TTC21A (Liu et al., 2019)] an estimated 50% or more of the molecular mechanisms behind MMAF are yet to be identified. Recently, a sporadic defect in the centriolar protein CEP135 was found to contribute to MMAF (Table 1). Sha et al. (2017) identified an inherited missense homozygous mutation in CEP135 in an infertile male with MMAF. Immunofluorescence analysis showed that CEP135 localized to the PC in normal spermatozoa, while the patient’s mutated proteins localized elsewhere in the sperm, forming ectopic aggregates in the sperm neck and flagella (Figure 2). Following ICSI, embryos demonstrated pronucleus formation and cleavage, but ultimately the mother failed to become pregnant. This suggests that a centriole abnormality can produce a phenotype that is exhibited later in embryogenesis.

#### Dysplasia of the Fibrous Sheath

The fibrous sheath is a structure surrounding the axoneme in the flagellar principle piece (Figure 2). Dysplasia of the fibrous sheath is a rare condition of immotile sperm, which have morphological deformities in the neck, midpiece, and tail and exhibit centriolar dysfunction. Microscopy of dysplastic sperm reveals an increased and disorganized fibrous sheath with disruption of the underlying axoneme (Chemes et al., 1987a). Immunofluorescence with the centriolar protein Centrin-1 shows frequent abnormal positioning of the centrioles, sometimes with duplication, resulting in two nucleus-implantation sites (Moretti et al., 2017). Initial attempts at ICSI with dysplastic sperm were unsuccessful at achieving pregnancy, due to failure of either fertilization or embryo development (Chemes and Rawe, 2003). Nakamura et al. (2005) used bovine oocytes for heterologous ICSI and found that the vast majority of injected oocytes showed no immunocytochemical evidence of sperm aster formation or cytoplasmic microtubule organization. This is consistent with the observation that only 2% of the dysplastic sperm expressed the centriolar protein centrin at the midpiece. Failure to form sperm asters in the setting of severely diminished centrin expression strongly suggests that the sperm centrioles are either abnormal or missing. Therefore, it seems likely that centriole abnormality is contributing to infertility in some of these patients.

#### Globozoospermia

Globozoospermia is a rare condition describing spermatozoa with a round head and disorganized mid-piece that lack both a functional acrosome and acrosomal enzymes (Singh, 1992). An absent acrosome prevents penetration of the oocyte zona pellucida, and was originally believed to be the sole cause of infertility in affected spermatozoa (Rybouchkin et al., 1996). The rate of successful fertilization with ICSI for men with globozoospermia is lower than the general ICSI success rate [reviewed in Dam et al. (2007)], suggesting that there may be additional problems with reproductive machinery that are not overcome by ICSI. Centriole dysfunction may help explain this discrepancy. Indeed, globozoospermic sperm appear to have centriole dysfunction, based on heterologous ICSI with bovine oocytes [reviewed in Dam et al. (2007)] (Table 1) as well as bio-fluorescent staining of Centrin-1 (Moretti et al., 2019). For example, Nakamura et al. (2002) found that globozoospermic sperm demonstrated a significantly lower rate of sperm aster formation compared to control fertile donor sperm, suggesting a centrosome dysfunction. However, these also showed a significantly lower rate of male pronucleus formation and a significantly higher rate of prematurely condensed chromosomes, suggesting a more pleiotropic mechanism. Many papers show that globozoospermic sperm centrioles have normal ultrastructure, but the composition has yet to be fully investigated. However, due to the heterologous nature of globozoospermia, the discovery of the atypical DC, and the functional data showing poor aster formation in globozoospermia embryos, we propose that more work is necessary to test whether or not malformed centrioles may be implicated in this disease.

### Asthenozoospermia

Poor sperm motility seen in asthenozoospermia impedes proper transport of spermatozoa to the oocyte for penetration and membrane fusion. Theoretically, this infertility should be completely resolved by ICSI since the spermatozoa are artificially transported to within the oocyte. However, the low rate of successful pregnancies with ICSI in the setting of complete asthenozoospermia suggests the presence of additional factors (Nagy et al., 1995; Casper et al., 1996; Kahraman et al., 1996; Nijs et al., 1996; Cayan et al., 2001; Fonttis et al., 2002; Chatzimeletiou et al., 2007; Terada et al., 2009). Immunocytochemical analysis of oocytes which had failed fertilization by an athenozoospermic donor showed several phenotypes, including two pronuclei without the presence of recognizable sperm aster formation, suggesting centrosomal dysfunction had caused fertilization arrest (Terada et al., 2009). Western blot and ELISA analysis found that the centriolar protein centrin and the centriolar and flagellar protein Tektin 2 are reduced in oligoasthenozoospermic men, suggesting that their lower levels can result in lower pregnancy percentage after ICSI (Hinduja et al., 2008, 2010; Bhilawadikar et al., 2013). Also, recently, the base of the sperm tail was found to function as an atypical centriole in the zygote (Avidor-Reiss and Fishman, 2018). Thus, centriolar dysfunction may not only impair sperm motility as originally believed but may also cause a disruption of zygotic intracellular processes that ICSI cannot remedy.

### Azoospermia Treated Using Round Spermatids

Azoospermia is defined as the complete absence of sperm from the ejaculate and is present in ∼15% of infertile men (Jarow et al., 1989). It can be treated using spermatids or testicular spermatozoa. However, the use of round spermatids has limited success, and fertilization rates were lower than those obtained using elongating spermatids or spermatozoa [discussed in Liu X.-Y. et al. (2012)]. One possible explanation is that the two sperm centrioles are not yet remodeled in round spermatids. How the status of centriolar remodeling affects embryo development needs further investigation.

### Unexplained Infertility

Unexplained infertility, the absence of any observable male or female factor, is known to cause infertility in 10–30% of cases (Hart, 2003; Gunn and Bates, 2016). Recently, Garanina et al. (2019) suggested that a longer sperm centriolar adjunct is associated with unexplained infertility. They found through transmission electron microscopy that the average total length of the PC and its centriolar adjunct was significantly longer in the spermatozoa of two unexplained infertility patients than in the spermatozoa of five healthy donors. The two affected patients displayed repeated zygotic arrest after *in vitro* fertilization. One patient had a centriolar adjunct nearly double the length seen in healthy donors and failed all fertility treatments. The second patient with intermediate findings (∼1.5 longer) finally conceived a healthy baby that was delivered at 40 weeks of gestation. Ultimately, the function of the centriolar adjunct is still unknown. In all mammals except for human, the centriolar adjunct is a transient structure present during spermiogenesis but absent in the mature spermatozoon (Figure 2). In human spermatozoa, the centriolar adjunct is present and was postulated to be characteristic of relative immaturity of the human sperm (Zamboni and Stefanini, 1971). Therefore, it is unclear if centriolar adjuncts are essential only in humans, if a long centriolar adjunct causes a problem, or if the long adjunct is a marker for other defects in the centrioles. Further study of the association between centriolar adjuncts and fertility outcome is needed.

## A Specific and Rapid Assessment Method of Sperm Centrioles is Needed

Several assays for studying typical sperm centrioles are available. However, the technology is either insufficiently specific or inappropriately laborious. Sperm cell centrioles has been assessed by electron microscopy, a laborious technique that is inadequate for large-scale studies and is inaccessible in most clinical settings (Chemes et al., 1987b; Moretti et al., 2016). Sperm centriolar protein content has been assessed by Western blot, which studied total protein and is not specific to the centriole and cannot conclusively implicate the centriole in infertility (Hinduja et al., 2010). Sperm centriole function has been assessed by microinjection of human sperm into bovine, rabbit, or hamster oocytes followed by immunofluorescence staining for aster formation, which has been useful in studying abnormal sperm centrioles in some infertility cases (Rawe et al., 2002; Terada et al., 2004; Yoshimoto-Kakoi et al., 2008). However, this method is now illegal according to the Dickey-Wicker amendment (the U.S. federal bill that prohibits funding for the creation of human embryos for research purposes). Because of these limitations, sperm centriole defect in infertile men has only been demonstrated in small case studies (Nanassy and Carrell, 2008; Chemes, 2012; Moretti et al., 2016; Sha et al., 2017). Therefore, there is a need for a specific and high-throughput method for assessing sperm centrioles and determining their association with certain embryonic phenotypes and outcomes.

## Future Directions

Previous animal and epidemiological studies of sperm centrioles provide a basis for a correlation between centriole abnormality and embryo development pathology; however, further investigation is needed for conclusiveness on this subject. This investigation should include new model mammals and more conclusive clinical research.

Since mice do not have detectable centrioles in the sperm and early embryo, other mammalian models with these centrioles should be developed. For instance, centriole inheritance in rabbits resembles that of humans, and they are suitable models because they are the smallest non-murine mammal with established genetic engineering (Liu et al., 2004; Terada, 2007). The generation of a model rabbit for studying centriole-based infertility would be very useful; however, to do so, the tools necessary for specifically interfering with the sperm centriole must first be identified. Characterizing the mechanisms of sperm centriole formation and function as well as their unique properties will provide the insights necessary to develop these tools. CRISPR/Cas9 editing can be used to alter sperm-specific sequences, which will enable the development of a specific model animal.

Despite the correlation between centriole dysfunction and infertility that has been proposed in the epidemiological literature, the extent and significance of this relationship is still not known. The main reason is that most human studies to date have not demonstrated a clear case of specific centriole dysfunction. Therefore, a diagnostic test should be developed to better identify sperm with specific centriolar defect. Also, because such defect is likely to affect only a small fraction of all infertility cases, the diagnostic test needs to be easy and rapid. One additional approach for improving epidemiological studies is to include advanced semen analysis that examines DNA and egg activation factors, followed by testing sperm that have only centriolar defect. Ultimately, these efforts should lead to more conclusive clinical research, such as large prospective cohort studies and randomized controlled trials.

Sperm centriole defect may be mediated by genetic, environmental, or infectious factors; however, no directed effort has been made to identify these factors. Therefore, there is a need to identify sperm centriole defect in human, to find their cause, and to characterize its impact on male infertility.

## Author Contributions

TA-R conceived, supervised, and wrote the manuscript. MM wrote the manuscript and searched the literature. EF prepared the figures. PS performed the clinical perspective.

## Conflict of Interest Statement

The authors declare that the research was conducted in the absence of any commercial or financial relationships that could be construed as a potential conflict of interest. The reviewer GC declared a past co-authorship with one of the authors TA-R to the handling Editor.
